# A Rare Case Report of Sitagliptin-Induced Angioedema

**DOI:** 10.7759/cureus.30077

**Published:** 2022-10-08

**Authors:** Nava R Sharma, Bharosa Sharma, Saral Lamichhane, Madalasa Pokhrel, Prajwal Shrestha

**Affiliations:** 1 Internal Medicine, Manipal College of Medical Science, Pokhara, NPL; 2 Internal Medicine, John H. Stroger, Jr. Hospital of Cook County, Chicago, USA; 3 Internal Medicine, Gandaki Medical College, Pokhara, NPL; 4 Internal Medicine, Montefiore Medical Center, New Rochelle, USA

**Keywords:** hereditary angioedema, c1 esterase inhibitor, diabetes mellitus, angioedema, sitagliptin

## Abstract

Sitagliptin, a dipeptidyl peptidase-4 inhibitor, is used for the treatment of type 2 diabetes mellitus. Sitagliptin-induced angioedema has increased with the simultaneous use of angiotensin receptor blockers and angiotensin-converting enzyme inhibitors. We present a rare case of a 50-year-old female diagnosed with sitagliptin-induced angioedema. On examination, she had both upper and lower lip swelling without any respiratory compromise. On further investigation, her C1 esterase inhibitor level was normal. After stopping sitagliptin, her symptoms resolved. Thus, cautious use of dipeptidyl peptidase-4 inhibitor is advised.

## Introduction

Sitagliptin is a drug used in patients with type 2 diabetes mellitus. It is a dipeptidyl peptidase (DPP-4) inhibitor that delays the inactivation of incretin hormones [1]. It is popular due to its good tolerability, low potential for hypoglycemia and neutral effect on weight. However, the side effects include an increased risk of pancreatitis and infections. Also, post-marketing analysis has shown rare hypersensitivity reactions such as anaphylaxis, angioedema, and exfoliative skin conditions [2]. Drug-induced angioedema is often sudden in onset and reversible after drug discontinuation. Drug-induced angioedema is commonly seen with the use of beta-lactam antibiotics, nonsteroidal anti-inflammatory drugs, and drugs blocking the renin-angiotensin-aldosterone system [3].

## Case presentation

A 50-year-old female came into the emergency department with a history of lip swelling for a day. She denied chest pain, dyspnea, or skin rashes. She had a history of treated breast cancer, seizure, asthma, and type 2 diabetes mellitus. Her medications included nifedipine, metformin, tamoxifen, levetiracetam, and sitagliptin. Sitagliptin was recently added to her treatment regimen. She had a history of two prior episodes of angioedema secondary to using angiotensin receptor blocker (ARB) and fosphenytoin, respectively. 

**Figure 1:**
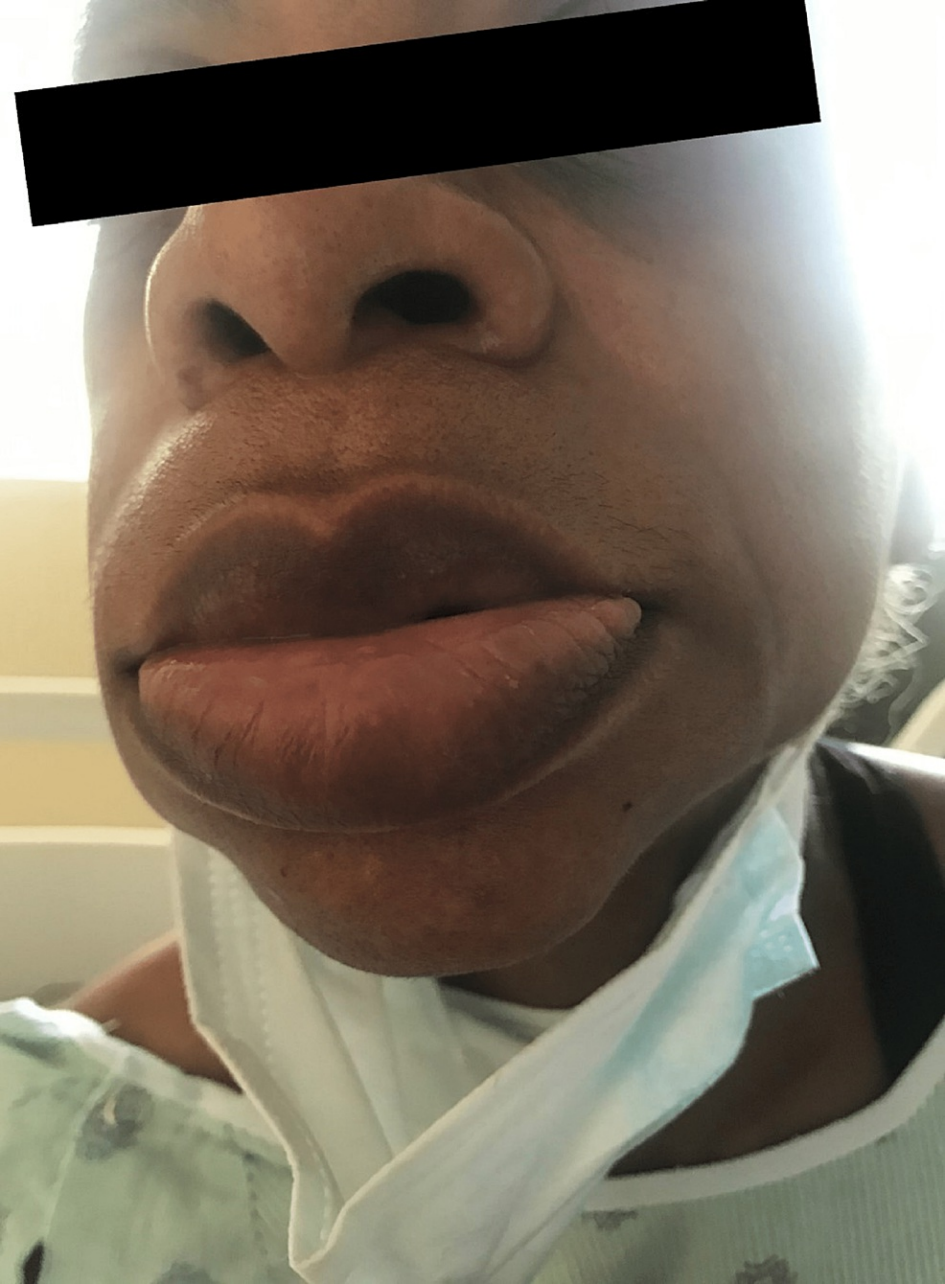
Sitagliptin-induced angioedema

On arrival, her blood pressure was 146/73 mmHg; her pulse rate was 69 per minute. Her oxygen saturation was over 95% on room air, with the respiratory rate at 18 breaths/minute. On examination, there was upper and lower lip swelling, as shown in Figure 1. There were no features of respiratory compromise on further assessment. 

Blood tests revealed normal blood count, liver function tests, and kidney function tests. In addition, the C1 esterase inhibitor level (C1INH) was also normal. 

She was treated with oral prednisone 40mg once a day and diphenhydramine 25mg per oral thrice a day for five days, after which her condition improved. Sitagliptin was stopped, and she was discharged home in stable condition. The patient came for a follow-up in a week with a complete resolution of her symptom.

## Discussion

Drug-induced angioedema is mainly associated with angiotensin-converting enzyme inhibitors (ACE-I). It is thought to be due to impaired bradykinin metabolism by the angiotensin-converting enzyme (ACE). Bradykinin is also a substrate for DPP-4, inhibiting sitagliptin [4]. Several studies published so far have elucidated angioedema from sitagliptin occurring with concurrent use of ACE-I or ARB [5,6]. One case report showed angioedema from sitagliptin recently started for diabetes without using an ACE-I or ARB, similar to our patient [7]. There is no clear consensus on the increased risk of angioedema in patients treated with sitagliptin. A large pooled analysis did not show an increased incidence of angioedema in patients who were treated with sitagliptin as compared to patients who were not exposed to sitagliptin, irrespective of ACE-I use [8].

As our patient had prior episodes of angioedema from an ARB and following administration of fosphenytoin for a seizure episode, this led to a suspicion that she could have an inherent defect in the pathway for bradykinin metabolism. But, both her previous C1INH level and a repeat C1INH level measured at this time were normal. In a similar case report previously discussed, a patient with a normal level of C1INH was noted to have a mutation for hereditary angioedema type 3 [7]. Our patient could also have a hereditary type 3 with episodes of angioedema being triggered by drugs such as ARB, sitagliptin, and fosphenytoin.

## Conclusions

Sitagliptin, a dipeptidyl peptidase-4 inhibitor, is used for the treatment of type 2 diabetes mellitus. Angioedema is a rare side effect of the drug. Hence, cautious use of DPP-IV inhibitors is warranted in patients with prior episodes of angioedema. Hereditary angioedema type 3 may be an important consideration in patients with normal C1INH levels.

## References

[1] Muscelli E, Casolaro A, Gastaldelli A (2012). Mechanisms for the antihyperglycemic effect of sitagliptin in patients with type 2 diabetes. J Clin Endocrinol Metab.

[2] (2011). Januvia (sitagliptin tablets) prescribing information. https://www.merck.com/product/usa/pi_circulars/j/januvia/januvia_pi.pdf.

[3] Inomata N (2012). Recent advances in drug-induced angioedema. Allergol Int.

[4] Brown NJ, Byiers S, Carr D, Maldonado M, Warner BA (2009). Dipeptidyl peptidase-IV inhibitor use associated with increased risk of ACE inhibitor-associated angioedema. Hypertension.

[5] Gosmanov AR, Fontenot EC (2012). Sitagliptin-associated angioedema. Diabetes Care.

[6] Cassano N, Nettis E, Di Leo E, Ambrogio F, Vena GA, Foti C (2021). Angioedema associated with dipeptidyl peptidase-IV inhibitors. Clin Mol Allergy.

[7] Arcani R, Martinez S, Gayet S (2017). Sitagliptin and angioedema. Ann Intern Med.

[8] Williams-Herman D, Engel SS, Round E (2010). Safety and tolerability of sitagliptin in clinical studies: a pooled analysis of data from 10,246 patients with type 2 diabetes. BMC Endocr Disord.

